# Medical Politics

**Published:** 1917-08-04

**Authors:** 


### The Hospital

The Workers' Newspaper of Administrative Medicine and Institutiona
Life, Administration, National Insurance and Health.

No. 1626, Vol. LXII. SATURDAY, AUGUST 4, 1917. PRICE ONE PENNY

### MEDICAL POLITICS.

The discussions which occupied the main attention of last week's Representative Meeting of the British Medical Association reveal an increasing conviction on the part of the profession that medical questions are likely in larger and larger measure to become the subject of popular attention and legislative activity. The promotion of health and the preservation of life are seen to be large national interests, and the war has pushed them into an acute prominence. Hence the certainty that in the immediate future Parliament and public opinion will insist upon a say in all matters which pertain to these ends. But few members of the profession are now prepared to stand on the severely individualistic platform and to claim that with the relations of doctor and patient no outside authority can have any concern. The movement of modern opinion is decidedly against this view, and the only open question is as to the extent to which professional activities ought to come under public survey. On this issue the doctors very naturally have much to say, and it cannot be expected that they will all speak with one and the same voice. Yet, to judge from the Representative Meeting, there are certain points on which the profession approaches unanimity. It is, for example, no longer in question that preventive medicine in its various operations must be administered by practitioners who are public officials. In large and populous areas this aim is indeed already fully achieved, and the work is in the hands of full-time officers who act under the direction of the local health authority. There still, however, remain districts in which the medical officer of health is engaged in private practice, with a consequent absence of the detachment from local pressure which such an office demands. But such a state of matters is a fading quantity, and there is a practically unanimous assent to the proposition that the care of the public health demands whole-time service with municipal or State control. Both the doctors and the laymen are in agreement on this point.

A different position is reached when the question of the individual sick man is raised. On paper it may be argued that with such an issue no public authority is concerned, and that the liberty of the subject demands the exclusion of such authority. But in the world of realities a claim of this order cannot stand upright. The community is undoubtedly concerned in the economic value and personal efficiency of its citizen units; individual illnesses may from their infectious nature be a source of danger to the whole civic organisation; and in some classes efficient medical aid cannot be obtained except by a scheme of organisation which the State alone can bring into being. These considerations form an immediate and overwhelming refutation of the argument that personal or curative medicine may be left to manage its own affairs in its own way. There is an even broader contribution to the same end. It is that many an individual illness brings to the surface the issue of ætiology, and such an inquiry at once relates to methods of prevention which may in practice involve matters of housing, of sanitary police, and of general working conditions. In some degree, therefore, what is commonly called private practice is certainly a subject of public concern, and it may not hope wholly to avoid public interest or even public interference. There are those who would arrange that this private and personal medicine should, equally with the operations of preventive medicine, be administered by State or municipal officials, and this is the basis of the policy known as a State Medical Service. The official doctor will be in every district, and the sick man must accept him just as he accepts the official sanitary officer; he has no opportunity for choice in the case of the latter, and equally he must welcome the doctor whom the State imposes.

Upon this point the Representative Meeting has spoken with no uncertain sound. To whole-time State medical officers charged with the duty of treating patients the profession will offer the strongest opposition. The prospect of a fixed salary, of regular hours, and of a retiring pension does not attract practitioners when these proposals involve the loss of the personal and intimate relations which at present exist between the family doctor and his patients, and which are of high material value for practical ends. This view came prominently to the surface in the discussions which took place relative to the establishment of a Ministry of Health. Such an organisation has been urged by the profession for many years, and it was supported at the recent meeting. But with practical unanimity it was claimed that the officers of the administration concerned with preventive medicine should be kept rigidly to their own department, and that officers having an equal status should be appointed for the supervision of clinical medicine or treatment so far as this is directed by public authority. The same attitude was displayed in the discussions on National Insurance. A minority still holds to the view that insurance patients ought to be left to make their own personal medical arrangements; but the majority of the profession recognises that what has been done cannot now be wholly undone, and that the area of practice covered by the principle of National Insurance is bound to increase. The doctors are determined to have their say upon the conditions which may be imposed by the State, and to preserve, alike for themselves and their patients, such a measure of freedom and independence as is compatible with the dignity of a liberal profession, while recognising at the same time the reasonable demands which are inseparable from the disbursement of public funds.

